# Supplementary material for: Aqueous Extract from Cuminum cyminum L. Seed Alleviates Ovalbumin-Induced Allergic Rhinitis in Mouse via Balancing of Helper T Cells
Source: Foods. 2022 Oct 15;11(20):3224. doi: 10.3390/foods11203224 (PMC9601982; doi:10.3390/foods11203224)
Supplement: Supplementary file 1 [file foods-11-03224-s001.zip › foods-1936440-supplementary.pdf]

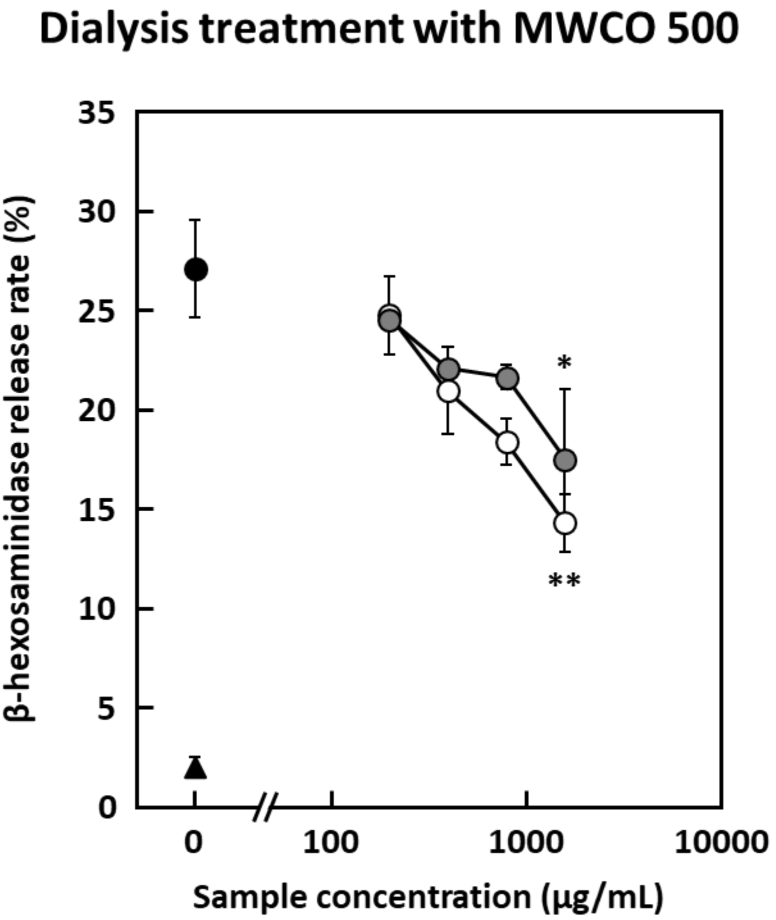

Figure S1. Effect of dialysis treatment on the anti-degranulation activity of cumin seed aqueous extract (CAE). Anti-dinitrophenyl (DNP) IgE-sensitized RBL-2H3 cells were treated with various concentrations of CAE or dialyzed CAE with degranulation induced by DNP- human serum albumin (HSA) stimulation. The control cells (closed circle) were treated with 10 mM sodium phosphate buffer as a vehicle and stimulated with DNP-HSA. The blank cells (closed square) were treated with 10 mM sodium phosphate buffer as a vehicle without DNP-HSA stimulation. Open circles and gray circles indicate CAE-treated cells and dialyzed CAE-treated cells, respectively. Data are expressed as means ± SEM of three independent experiments. *$p < 0.05$ and **$p < 0.01$ against control cells by Tukey test.
